# Long-term survival of patients suffering from glioblastoma multiforme treated with tumor-treating fields

**DOI:** 10.1186/1477-7819-10-220

**Published:** 2012-10-24

**Authors:** Aaron Michael Rulseh, Jiří Keller, Jan Klener, Jan Šroubek, Vladimír Dbalý, Martin Syrůček, František Tovaryš, Josef Vymazal

**Affiliations:** 1 Department of Radiology, Na Homolce Hospital, Prague, Czech Republic; 2 Charles University in Prague, 3rd Medical Faculty, Prague, Czech Republic; 3 Department of Neurosurgery, Na Homolce Hospital, Prague, Czech Republic; 4 Department of Pathology, Na Homolce Hospital, Prague, Czech Republic; 5 Department of Neurology, Charles University in Prague, 1st Medical Faculty, Prague, Czech Republic

**Keywords:** Glioblastoma multiforme, Recurrent glioblastoma multiforme, Tumor-treating fields, Long-term survival

## Abstract

Glioblastoma multiforme (GBM) is the most common and malignant primary intracranial tumor, and has a median survival of only 10 to 14 months with only 3 to 5% of patients surviving more than three years. Recurrence (RGBM) is nearly universal, and further decreases the median survival to only five to seven months with optimal therapy. Tumor-treating fields (TTField) therapy is a novel treatment technique that has recently received CE and FDA approval for the treatment of RGBM, and is based on the principle that low intensity, intermediate frequency electric fields (100 to 300 kHz) may induce apoptosis in specific cell types. Our center was the first to apply TTField treatment to histologically proven GBM in a small pilot study of 20 individuals in 2004 and 2005, and four of those original 20 patients are still alive today. We report two cases of GBM and two cases of RGBM treated by TTField therapy, all in good health and no longer receiving any treatment more than seven years after initiating TTField therapy, with no clinical or radiological evidence of recurrence.

## Background

Glioblastoma multiforme (GBM) is the most common and malignant primary intracranial tumor, representing as much as 30% of primary brain tumors with increasing incidence in some geographic regions [1]. Its incidence has been shown to increase with age [2]. Despite the introduction of aggressive treatment with temozolomide, the median survival time of adult patients remains approximately 10 months and as high as 14 months in patients receiving combined treatment with radiotherapy [3]. Only 3 to 5% of patients survive more than three years [4], and sporadic reports of survival exceeding five years are rare [5]. The exact clinical and molecular factors that contribute to such long-term survival are still unknown; however, younger age and a high Karnofsky performance scale (KPS) are considered prognostically favorable factors [4]. Recently, MGMT gene promoter methylation and IDH1 mutation have been shown to correlate with longer survival as well [6]. Recurrence of GBM is nearly universal, and patients with recurrent glioblastoma multiforme (RGBM) fare even worse, with a median survival of only five to seven months with optimal therapy [7].

Tumor-treating fields (TTField) therapy is a novel treatment technique with the potential to treat various forms of cancer. TTField therapy is based on the principle that low intensity, intermediate frequency electric fields (100 to 300 kHz) have an anti-mitotic effect which acts during late metaphase and anaphase, with specific frequencies affecting specific cell types [8]. The applied fields disrupt mitotic spindle microtubule assembly and the segregation of intracellular organelles during cell division, leading to apoptosis [9]. TTField therapy has been tested in patients with advanced non-small cell lung cancer [10] and has recently received CE and FDA approval for the treatment of RGBM based on the results of a phase III clinical trial [11].

Our center was the first in the world to apply TTField treatment to histologically proven GBM patients in a small pilot study of 20 individuals in 2004 and 2005 (Table 1). The inclusion criteria of the study included a KPS ≥70% and age ≥18 years, and the patients were divided into two groups. The first group consisted of 10 patients diagnosed with RGBM after failing temozolomide treatment that were treated with TTField therapy alone [9]. The second group consisted of 10 newly diagnosed GBM patients at least four weeks post radiation therapy (with adjuvant temozolomide) [12] that received TTField therapy combined with maintenance temozolomide. The treatment duration in individual patients varied between one and one and a half years, and all histological samples were independently examined in two laboratories in two countries. We report two cases of GBM and two cases of RGBM treated by TTField therapy, all in good health and no longer receiving any treatment more than seven years after initiating TTField treatment, with no clinical or radiological evidence of recurrence.

**Table 1:** Participant baseline characteristics

**Patient no.**	**Group**	**Date of birth**	**Date of inclusion**	**Age at inclusion**	**Gender**	**Weight (kg)**	**KPS (%)**	**Tumor location**
1	RGBM	07/1950	05/2004	53.8	Male	70	100	R. Temporoparietal
2	RGBM	08/1945	05/2004	58.8	Male	83	100	R. Temporal
3*	**RGBM**	**06/1952**	**08/2004**	**52.2**	**Female**	**65**	**70**	**R. Parietal**
4*	**RGBM**	**08/1961**	**08/2004**	**43.1**	**Male**	**85**	**100**	**L. Frontoparietal**
5	RGBM	05/1937	10/2004	67.5	Male	83	80	R. Temporoparietal
6	RGBM	05/1953	01/2005	51.7	Male	103	70	R. Parietooccipital
7	RGBM	04/1950	01/2005	54.8	Male	85	90	R. Frontotemporal
8	RGBM	06/1966	06/2005	39	Male	72	100	R. Temporoparietal
9	RGBM	06/1977	08/2005	28	Female	70	90	L. Temporoparietooccipital
10	RGBM	08/1948	09/2005	57	Female	68	70	L. Temporal
11	GBM	11/1968	01/2005	36	Male	77	100	L. Frontal
12	GBM	04/1935	05/2005	70	Male	79	90	R. Temporal
13*	**GBM**	**12/1973**	**10/2005**	**32**	**Male**	**85**	**100**	**R. Frontal**
14	GBM	03/1948	01/2006	58	Male	81	100	R. Temporal
15	GBM	10/1963	01/2006	42	Male	96	90	R. Temporal
16	GBM	03/1961	01/2006	45	Female	50	100	R. Frontal
17*	**GBM**	**08/1973**	**04/2006**	**33**	**Female**	**65**	**90**	**R. Frontal**
18	GBM	07/1951	10/2006	55	Male	80	100	L. Occipital
19	GBM	05/1941	09/2006	65	Male	85	80	L. Frontotemporal
20	GBM	05/1951	01/2007	56	Male	82	90	R. Temporal

* case in present report.

Baseline characteristics of all 20 participants in the original pilot study. Dates are presented as month/year for simplicity, age calculations were performed on exact dates.

GBM, glioblastoma multiforme (newly diagnosed); KPS, Karnofsky performance scale; L., left; No., number; R., right; RGBM, recurrent glioblastoma multiforme.

## Case presentation

### Case 1

**Figure 1:**
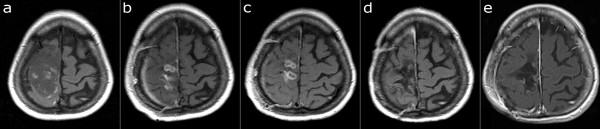
**Serial MR imaging in Case 1.** T1-weighted image after application of contrast agent. **a**) April 2004, before surgery. GBM located in the right central region. **b**) July 2004, post-operative radiotherapy and chemotherapy. Two enhancing lesions present. **c**) September 2004, one month after the start of TTField therapy. The dorsal enhancing lesion increased in size, highly suspicious of tumor recurrence. **d**) June 2005, TTField treatment. No enhancing lesion present. **e**) August 2011. No enhancing lesion present. GBM, glioblastoma multiforme; MR, magnetic resonance; TTField, tumor-treating fields.

A 52-year-old woman with a history of epileptic seizures and left-sided hemiparesis was diagnosed with an intra-axial brain tumor, suspected to be a high-grade glioma based on the magnetic resonance imaging (MRI) findings (Figure 1a). The tumor was resected with the help of functional blood oxygen level dependence (BOLD-fMRI) neuronavigation in April 2004. Gross total resection was performed and pathological analysis revealed clear evidence of glioblastoma. Standard radiotherapy (60 Gy) and chemotherapy with temozolomide followed. After radiotherapy and chemotherapy, a follow-up MRI in July 2004 showed two enhancing lesions that were highly suspected to be tumor recurrence (Figure 1b). TTField therapy was initiated in August 2004 as monotherapy. In September 2004, one month after starting TTField therapy, one of the enhancing lesions increased in size (Figure 1c); however, treatment with TTField therapy was continued since the progression was asymptomatic. By February 2005, both enhancing lesions had disappeared (Figure 1d) and are no longer detectable (Figure 1e). TTField treatment was discontinued after one year in August 2005, with no treatment administered after that time. The last MR examination from August 2011 shows no evidence of enhancing tumor. The patient has mild, residual left-sided hemiparesis, and otherwise feels completely healthy with no subjective complaints and a KPS of 90.

### Case 2

**Figure 2:**
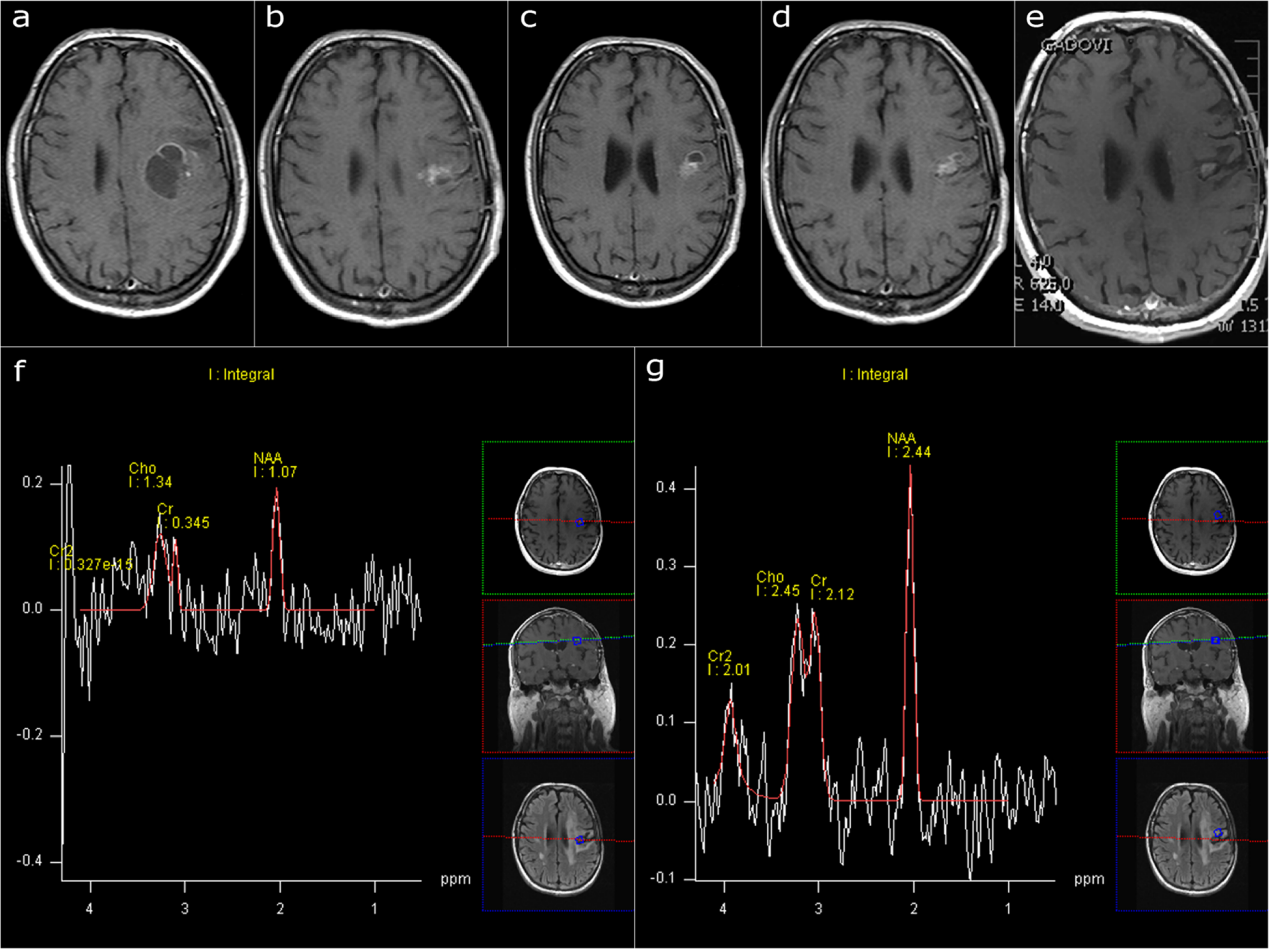
**Serial MR imaging in Case 2.** T1-weighted image after application of contrast agent. **a**) January 2004, before surgery. GBM located in the left frontal region. **b**) August 2004, post-operative radiotherapy and chemotherapy. Start of TTField treatment. An enhancing lesion suspected to be recurrent or residual tumor. **c**) March 2005, TTF treatment. The enhancing lesion became partly cystic. **d**) June 2005, regression of the cystic part. A subtle enhancing lesion still present. **e**) November 2011. A subtle enhancing lesion without progression. **f**) Proton MR spectroscopy of the enhancing lesion with dominant noise signal, suggesting gliosis rather than tumor. **g**) Neighboring spectrum is practically normal, demonstrating that MR spectroscopy provided reliable data from the selected slice. GBM, Glioblastoma multiforme; MR, magnetic resonance; TTField, tumor-treating fields.

A 41-year-old male presented with pronunciation difficulties in December 2003. Neurological examination revealed slight right-sided hemiparesis, and MRI revealed an intra-axial tumor suspected to be high-grade glioma (Figure 2a). Surgery was delayed due to intercurrent infection (influenza) until March 2004, and the tumor was then partially resected. Pathological analysis revealed histological characteristics of glioblastoma. Standard radiotherapy (60 Gy) and chemotherapy with temozolomide followed. A follow-up examination in August 2004 showed an enhancing lesion suspected to be a recurrent tumor (Figure 2b). TTField therapy was initiated in August 2004. By March 2005 the enhancing tumor had progressed and become cystic (Figure 2c). Again, treatment with TTField therapy was continued due to the asymptomatic nature of this progression. By October 2005 the tumor had regressed (Figure 2d) while the patient was still receiving TTField therapy. TTField therapy was discontinued in February 2006. A discrete, enhancing lesion is still present (last MRI in November 2011, Figure 2e). This small, enhancing lesion was examined with MR spectroscopy (Figure 2f) and showed noise-signal only, while the spectra in neighboring voxels were practically normal (Figure 2g). Positron emission tomography (PET) did not reveal any tumor-like patterns. The patient is in good health, has minor difficulties with speech, and is completely independent, with a KPS of 90 to 100.

### Case 3

**Figure 3:**
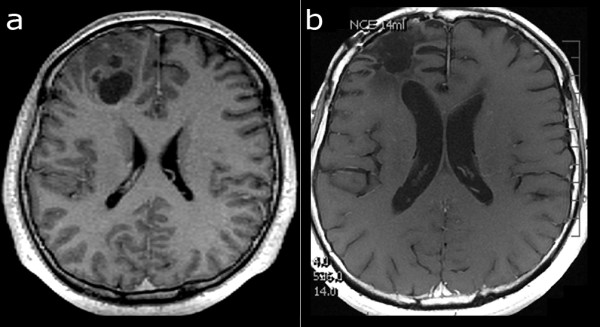
**Serial MR imaging in case 3.** T1-weighted image after application of contrast agent. **a**) January 2005 before surgery. GBM located in the right frontal region. **b**) December 2011. No tumor recurrence detected. GBM, glioblastoma multiforme; MR, magnetic resonance.

A 31-year-old male presented with an epileptic seizure in January 2005. MRI examination revealed a tumor in the right frontal lobe that was suspected to be a high grade glioma (Figure 3a). The tumor was totally resected macroscopically (gross total resection) and showed clear histopathological characteristics of glioblastoma (World Health Organization (WHO) grade IV). Standard radiotherapy (60 Gy) and chemotherapy with temozolomide followed. TTField treatment was started in October 2005 concomitant to maintenance temozolomide and both treatments were discontinued in October 2006. Since that time, no tumor recurrence has been detected (Figure 3b). The patient is in good health, off all treatment and with a KPS of 100.

### Case 4

**Figure 4:**
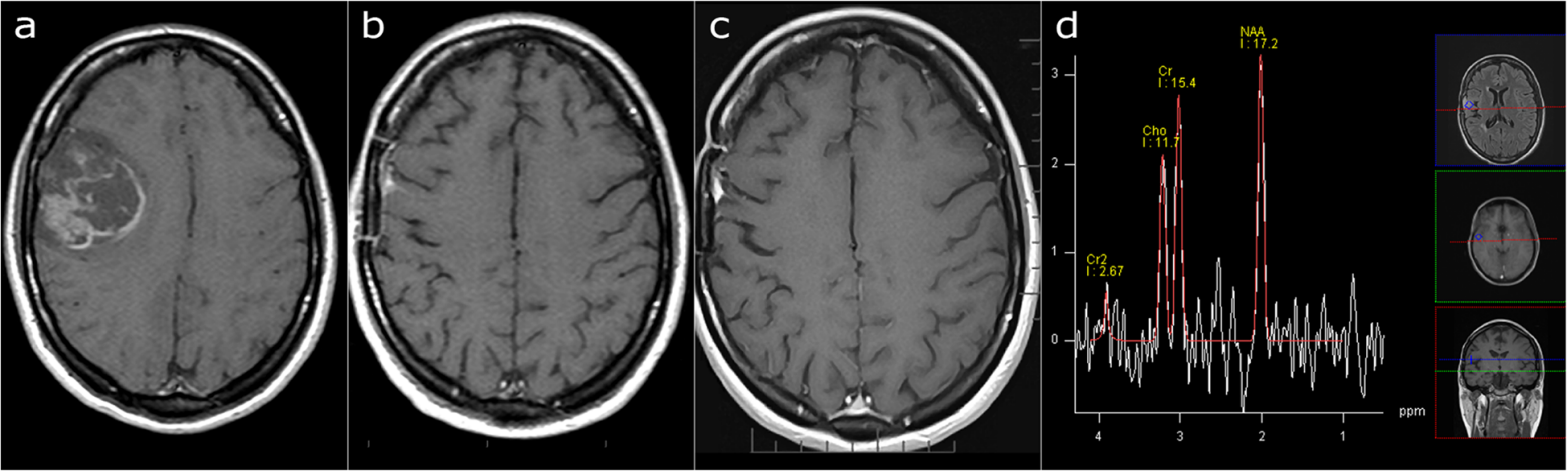
**Serial MR imaging in case 4.** T1-weighted image after application of contrast agent. **a**) November 2005, before surgery. GBM located in the right frontal region. **b**) May 2006, a small, extra-axial enhancing lesion. **c**) September 2011, no change of the enhancing lesion. **d**) FLAIR and MR spectroscopy images, September 2011. The small volume of increased signal intensity on FLAIR images did not show a tumor-like pattern on MR spectroscopy. FLAIR, Fluid Attenuated Inversion Recovery; GBM, glioblastoma multiforme; MR, magnetic resonance.

A 33-year-old female underwent MRI of the brain following an epileptic seizure in November 2005. A tentative diagnosis of high-grade glioma was made based on MRI findings (Figure 4a). The tumor was completely resected macroscopically (gross total resection) in February 2006 and showed clear histopathological characteristics of glioblastoma (WHO grade IV). Standard radiotherapy (60 Gy) and chemotherapy with temozolomide followed. No recurrence was noted on follow-up MRI in February 2007 (Figure 4b). TTField treatment was started in April 2006 concomitant to maintenance temozolomide and both treatments were discontinued in April 2007. No tumor recurrence has been detected on a number of follow-up MRI examinations (Figure 4c), the last of which was performed in September 2011. MR spectroscopy in a small volume of tissue with corresponding increased signal intensity on Fluid Attenuated Inversion Recovery (FLAIR) images did not show a tumor-like pattern (Figure 4d). Currently the patient is off all treatment, in good health, with a KPS of 100.

## Discussion

Despite multi-modal treatment, the prognosis of GBM remains poor. Recurrence is likely inevitable provided the patient survives long enough, and further reduces the median survival to only five to seven months [4,7]. There have been documented cases of GBM patients surviving longer than three years, termed long-term survival (LTS), representing approximately three to five percent of GBM patients [4]. Survival of GBM patients longer than five years, however, is exceptional, representing as few as 0.5% of patients [5].

**Figure 5:**
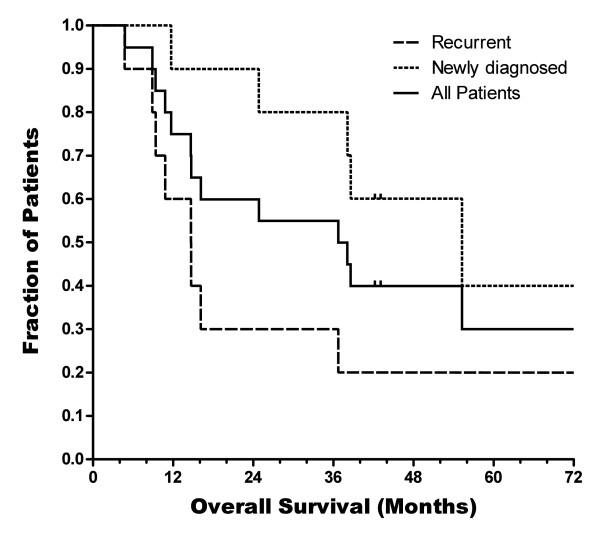
**Kaplan-Meier survival curves.** Kaplan-Meier survival curves for all 20 participants in the original pilot study, both as a single group and divided into recurrent and newly diagnosed glioblastoma multiforme. Two patients were lost to follow-up in the newly diagnosed group and are represented by censor marks.

Twenty percent of the participants in our pilot study (4 out of 20) have survived until the time of this report, roughly seven years (Figure 5). These individuals continue to undergo regular neurological and radiological examinations, and do not show any signs of recurrence. The data from standard MR imaging are further supported by MR spectroscopy that does not show any tumor-like patterns in regions with corresponding abnormal signal intensity.

Younger age and a higher KPS have been proposed as prognostically favorable parameters for longer survival [4]. The mean age of our LTS-RGBM patients (Cases 1 and 2) was 47.5 years compared to 51.5 years for the rest of the RGBM group [9]. This difference is not striking. Although we cannot completely exclude pseudo-progression or radiation necrosis in these patients diagnosed without histological verification of the recurrent lesion, their continued survival is still remarkable. In the group of newly diagnosed GBM, the mean age of LTS-GBM patients (Cases 3 and 4) was 32 years compared to 51 years for the rest of the group. This was likely a contributing factor to their long term survival; however, this does not explain seven years of disease-free survival. All of the patients in the trial had a KPS ≥70 and the median KPS was 80.

TTField therapy has been shown to effectively inhibit glioma cell replication *in vitro* and *in vivo*[8,9]. The published results of the pilot trial using TTField therapy in GBM patients were extremely promising and served as the basis for a phase III clinical trial comparing TTField therapy to the best available active chemotherapy in patients with RGBM [11]. The phase III trial showed that patients with RGBM had comparable overall survival to those receiving chemotherapy without the side effects of chemotherapy and with a better quality of life. In the present study, no serious, probable, treatment-related adverse events occurred, with only contact dermatitis treated by topical corticosteroid documented in 17 of 20 patients. In the phase III trial, 8% of TTField therapy patients survived for longer than three years [11]. The reasons for the smaller number of long term survival patients in the phase III trial compared to the pilot trial is likely related to the younger age of the patients presented in this report, the fact that they were at their first recurrence after temozolomide (versus second to third recurrence in the phase III trial) and, most importantly, continued TTField therapy for many months, despite initial growth of the contrast enhancing lesion while on therapy. Thus, we suggest that in order to increase the probability of response to TTField therapy and subsequent long term survival, TTField treatment should be continued even in the face of initial radiologic tumor growth.

## Conclusions

In the present paper we report two cases of GBM and two cases of RGBM treated by TTField therapy, all in good health and no longer receiving any treatment more than seven years after initiating TTField therapy, with no clinical or radiological evidence of recurrence. Our results indicate that TTField treatment may be remarkably successful in a subgroup of GBM/RGBM patients, and further investigation is needed to identify any unique characteristics of this patient group.

## Consent

Written informed consent was obtained from all patients for publication of this case report and any accompanying images. A copy of the written consent is available for review by the Editor-in-Chief of this journal.

## Abbreviations

BOLD-fMRI: Blood oxygen level dependent functional magnetic resonance imaging; FLAIR: Fluid Attenuated Inversion Recovery; GBM: Glioblastoma multiforme; KPS: Karnofsky performance scale; LTS: Long-term survival; MRI: Magnetic resonance imaging; PET: Positron emission tomography; RGBM: Recurrent glioblastoma multiforme; TTField: Tumor-treating fields; WHO: World Health Organization.

## Competing interests

JV, JS, VD and FT are consultants for NovoCure, Ltd, Haifa, Israel. AMR, JiK, JaK and MS have no competing interests to declare.

## Authors’ contributions

JV participated in conception and design of the manuscript; acquisition, analysis and interpretation of the data; initial drafting and revision of the manuscript; and approval of the final version. AMR participated in conception and design of the manuscript, drafting and revision of the manuscript, preparation of images and approval of the final version. JiK participated in conception and design of the manuscript, drafting and revision of the manuscript and approval of final version. JaK, JS, VD, MS and FT participated in the acquisition, analysis and interpretation of the data, revision of the manuscript and approval of the final version. All authors read and approved the final manuscript.
